# Asymmetric Spread Analysis of Heart Rate Variability in XC Mountain Biking During a 20-Minute Autonomic Profile Test

**DOI:** 10.3390/s25154677

**Published:** 2025-07-29

**Authors:** Luis Javier Tafur-Tascón, María José Martínez-Patiño, Yecid Mina-Paz

**Affiliations:** 1 Grupo de investigación MEDES, Institución Universitaria Escuela Nacional del Deporte, Cali 76001, Colombia; luis.tafur@endeporte.edu.co; 2 Facultad de Ciencias de la Educación y Del Deporte, Universidad de Vigo, 36310 Vigo, Spain; mariajosemartinezpatino@gmail.com; 3 Departamento de Medicina Física y Rehabilitación, Facultad de Salud, Escuela de Medicina, Universidad del Valle, Cali 76001, Colombia; 4 Facultad de Educación a Distancia y Virtual, Institución Universitaria Antonio José Camacho, Cali 76001, Colombia

**Keywords:** autonomic nervous system, mountain biking, heart rate variability, asymmetry

## Abstract

The heart is innervated by the autonomic nervous system (ANS), which plays a role in regulating the heart rate. Cross-country mountain biking (MTBXC) is a sport with high physiological demands, where the autonomic nervous system plays a significant role. The main objective of this study was to analyze the asymmetry of heart rate in Colombian National Team mountain bikers, sub-23 category, during a 20 min cardiovascular autonomic profile test. Method: The cardiovascular autonomic profile was measured through heart rate variability during a 20 min test, divided into eight phases (supine, controlled ventilation at 10 cycles/min, controlled ventilation at 12 cycles/min, postural change, orthostasis, Ruffier test, 1 min recovery, and final recovery) in a group of n = 10 MTB cyclists from the National Sub-23 Team, including 5 males and 5 females. Results: The results for the male athletes were as follows: age: 19 ± 1 years; VO2max: 67.5 mL/kg/min; max power: 355 W; HRmax: 204 bpm. The results for the female athletes were as follows: age: 19 ± 1 years; VOmax: 58.5 mL/kg/min; max power: 265 W; HRmax: 194 bpm. Both genders showed the expected autonomic behavior in each phase. Asymmetrical propagation of heart rate was observed, with a greater deceleration pattern after postural changes and effort and a symmetrical acceleration pattern in these two phases. Discussion: Athletes exhibit increased vagal response compared to non-athletes. Mountain bikers show rapid heart rate reduction after exertion. Conclusion: This study demonstrates how mountain bikers exhibit increased heart rate deceleration following sympathetic stimuli.

## 1. Introduction

The heart is innervated by the autonomic nervous system (ANS), which plays an important role in heart rate regulation [1]. Mountain biking, specifically cross-country (MTBXC), is an Olympic sport characterized by cyclic, intermittent, high-risk features with changing courses and high physiological demands, where the autonomic nervous system is essential to meet these demands. Heart rate variability (HRV) is the physiological variation in the time interval between two heartbeats, resulting from the modulation exerted by the autonomic nervous system on the heart [2]. Therefore, HRV reflects the behavior of the autonomic nervous system (ANS), representing the control that is exerted by the sympathetic or parasympathetic nervous system in the physiological adaptations that are necessary for different situations. Measurement of HRV can be carried out using two methods: time domain analysis, which considers cardiac periods using statistical tools, and frequency domain analysis, which analyzes heart rate oscillations. The asymmetry of heart rate refers to the quality of asymmetry, the distribution of accelerations, and how acceleration and deceleration patterns of heartbeats are distributed.

Competitive mountain biking, especially in the cross-country (MTBXC) discipline, imposes a unique set of physiological demands that challenge the autonomic system of athletes. In mountain bike races, cyclists face constant pace changes (explosive efforts on technical climbs followed by brief descents), leading to rapid autonomic transitions, as well as additional stress due to irregular terrain and technical factors [3]. Comparative studies have indicated that, at equal external load, mountain biking generates greater adrenergic responses than road cycling due to these technical and emotional factors: the need to maneuver over obstacles, jumps, and descents frequently triggers adrenaline surges, intensely activating the sympathetic nervous system (SNS) [1,3,4,5].These abrupt sympathetic activations result in rapid increases in heart rate and blood pressure during competition, followed by periods in which the athlete must catch their breath and lower their heart rate as much as possible before the next effort. Therefore, the ability of a mountain biker to effectively alternate between sympathetic activation and vagal recovery is crucial for their performance.

HRV plays a prominent role as an indicator of how a cyclist’s body adapts to these repetitive demands [6]. High HRV at rest or during nighttime, throughout a training season, is often correlated with a good recovery state and favorable cardiovascular adaptation to intense aerobic training. Among elite U-23 cyclists, who typically accumulate high training volumes while their bodies are still maturing, HRV monitoring allows for early detection of overload signs. For example, sustained reductions in parameters such as RMSSD or HF over a week of training may indicate a state of accumulated fatigue or overreaching, signaling the need to introduce rest days or lighter workloads [5].

The literature reports that after extreme competitive efforts, the HRV can drop acutely and take more than 24 h to return to baseline values, reflecting a state of residual autonomic tension. In a study with mountain bikers, significant changes in HRV indices were observed after each stage of a 3-day race, accompanied by an inability of the heart rate and heart rate variability to fully recover even after 24 h of rest [4,5]. This evidence suggests that stage race competitions impose a cumulative stress on the ANS, which must be considered when planning recovery. Consequently, monitoring HRV before, during, and after competitions or demanding training sessions is used to guide recovery strategies for these athletes. An incomplete recovery of the vagal component of HRV suggests the need for additional rest, while a prompt normalization indicates that the cyclist is ready for new stimuli. HRV acts as an internal thermometer of the physiological stress load, allowing for better training periodization to maximize adaptations and performance [7].

Regarding Heart Rate Asymmetry (HRA), its relevance in the adaptation of mountain bikers is still being explored. Given that this sport involves frequent sudden accelerations (short sprints, uphill starts) followed by decelerations (brief stops, technical descents), the hypothesis is that a well-trained autonomic system could exhibit a certain favorable asymmetry [8]. This could be reflected in a Porta Index > 50% or an elevated Guzik% during post-exercise recovery phases, indicating that the athlete induces greater variability through decelerations (vagal activity) than through accelerations, facilitating recovery [9]. Although few studies have directly addressed HRA in cyclists, findings from endurance athletes can be extrapolated: in highly trained athletes, there is a tendency for greater contribution of heart rate decelerations following an orthostatic or exercise stimulus compared to less trained individuals. In practice, this would mean that a well-adapted mountain biker would show a more pronounced heart rate drop (and associated variability) at the end of an intense segment compared to a less fit cyclist whose heart remains more accelerated and with reduced variability. Such differences in heart rate asymmetry (HRA) could help identify athletes with superior autonomic recovery capacity, regardless of their absolute resting HRV values [10]. For young U-23 cyclists, this is valuable for monitoring their development: ideally, as training takes effect, not only will their physical performance increase, but their autonomic profile should also become more efficient, manifested in robust HRV and perhaps in specific asymmetry patterns that are indicative of vagal control maturation.

There is limited evidence regarding changes in heart rate asymmetry in athletes and its association with autonomic regulation. Therefore, the main objective of this study was to analyze heart rate asymmetry in professional Colombian mountain bikers, sub-23 category, through a 20 min autonomic test.

## 2. Methodology

### Autonomic Profile Test

The measurement of the cardiovascular autonomic profile was conducted through a heart rate variability analysis using a 20 min test, divided into 8 phases [11]: supine position (5 min) (Phase 1), controlled breathing at 10 cycles/min (VC10) (1 min) (Phase 2), controlled breathing at 12 cycles/min (VC12) (1 min) (Phase 3), postural change (1 min) (Phase 4), orthostatism (3:15 min) (Phase 5), Ruffier test (0:45 seg) (Phase 6), 1 min recovery (1 min) (Phase 7)*,* and final recovery (supine position (5 min) (Phase 8). The study was performed on a group of n = 10 mountain bikers from the U-23 COLOMBIA National Team, consisting of male (n = 5) and female (n = 5) athletes over 18 years old. These athletes were required to have participated in national and international competitions, have prior experience with the test, have been training continuously for at least three years, for three hours per day and five days per week. Participants were instructed not to train, consume alcohol, or use sympathomimetic substances within 24 h prior to the test. After the test, Performance indicators were characterized by measuring direct oxygen consumption using ergo spirometer k5 by COSMED. A maximal incremental test was applied as follows: (70 W/30 W/1 min), VO_2max_, maximal power, and maximal heart rate was computed.

The study was conducted as part of the training program under the supervision of the team’s coaches. All subjects received both verbal and written information regarding potential risks and benefits and signed an informed consent form. This research project was approved by the Institutional Human Ethics Review Committee of the National School of Sport under protocol No. 40.07.204. The participants attended the Exercise Physiology Laboratory at the IPS of the “Institución Universitaria Escuela Nacional del Deporte” to undergo the autonomic function test. The test was performed in a closed room with a temperature between 22 °C and 24 °C and a relative humidity between 50% and 60% in all trials. A qualified sports medicine specialist, an expert physiologist in the test, and a Ph.D. student in Education, Sport, and Health from the University of Vigo—all with experience in this type of test—were present. The laboratory was adequately equipped to handle any potential clinical events. The participants’ body weight was recorded using a Tanita BC-585F FitScan^®^ body composition monitor, and their height was measured using a CE 0123 UK DRY stadiometer upon arrival at the laboratory. The beat-to-beat RR interval time series was recorded for all phases using a Polar Vantage V2 (Polar Electro Oy, Kempele, Finland) in conjunction with a Polar H10 (Polar Electro Oy) heart rate chest belt monitor, which has been reported to be highly accurate in detecting RR intervals at rest and during exercise.

## 3. Data Analysis

The recorded data were analyzed using Kubios HRV (Preminum version 3.3.1, Kuopio, Finland®), where temporal domain variables such as RmSSD and pNN50 were computed. Through Fast Fourier Transform (FFT) for the frequency domain analysis, the power values of high frequencies (HFs—0.15–0.4 Hz ms²), low frequencies (LFs—0.04–0.15 Hz ms²), and very low frequencies (VLFs, ms²) were recorded, along with the LF/HF ratio for each phase of the test.

The deltas (∆RR) for each phase were calculated (Y = X₂ − X₁), and subsequently, the asymmetric spread propagation analysis of heart rate variability was performed by computing the ASI (Asymmetric Spread Index) from all Poincaré plots based on the method proposed by Rohila and Sharma [12] the following indices of HRA were calculated using Python 3.12 (Python Software Foundation, 2023) [13] and PyBios [14].

### 3.1. Calculation of the Asymmetric Spread Index (ASI)

The ASI was computed from all Poincaré plots, following the mathematical definition proposed by Rohila and Sharma:

$$\sqrt{\frac{\sum_{j=1}^{m}\frac{(a_{j}-\bar{a}{)}^{2}}{m}}{2\times \sum_{k=1}^{m+n}\frac{(a_{k}-\bar{a}{)}^{2}}{m+n}\;}}$$

where

(1) $$\bar{a}=\frac{\sum_{j=1}^{m}a_{j}}{m}$$

m: The total number of acceleration points (i.e., points above the identity line LI).

n: The total number of deceleration points (i.e., points below the identity line LI).

aj: The individual acceleration differences above the identity line (LI) in the Poincaré plot.

**Equation (1). Asymmetric Spread Index formula.**

The formula indicates the standard deviation of the arc of each point above the identity line, divided by the double standard deviation of the arc for all points. According to the literature, an ASI value close to 50% (symmetric) indicates a balanced response of sympathetic and parasympathetic activity [12].

### 3.2. Porta’s Index (PI%)

Porta’s Index measures the proportion of heartbeats where the next R-R interval is shorter than the previous one (i.e., accelerations). A higher PI% indicates more accelerations relative to decelerations [15].

(2) $$PI\%=\frac{N\left(∆RR^{-}\right)}{N(∆RR\neq 0)}\cdot 100$$

**Equation (2). Porta’s Index formula.**

### 3.3. Guzik’s Index (GI%)

Guzik’s Index quantifies the magnitude of asymmetry by comparing the squared distances of points above and below the identity line in the Poincaré plot. A higher GI% means stronger accelerations compared to decelerations [16].

(3) $$GI\%=\frac{\sum_{i=1}^{N(∆RR^{+})}∆RR^{+}(i{)}^{2}}{\sum_{i=1}^{N(∆RR)}∆RR(i{)}^{2}}\cdot 100$$

**Equation (3). Guzik’s Index formula** **.**

### 3.4. Ehler’s Index (EI)

Ehler’s Index assesses the skewness of R-R interval differences. EI > 0: Skewed toward accelerations. EI < 0: Skewed toward decelerations [17].

(4) $$EI=\frac{\sum_{i=1}^{N(∆RR)}∆RR(i{)}^{3}}{{\left(\sum_{i=1}^{N(∆RR)}∆RR(i{)}^{2}\right)}^{3/2}}$$

**Equation (4). Ehler’s Index formula**.

where
Δ(RR): The difference between two consecutive R-R intervals (ΔRR = RRₙ₊₁ − RRₙ).
Δ(RR⁻): The negative values of Δ(RR), representing heart rate accelerations.
Δ(RR⁺): The positive values of Δ(RR), representing heart rate decelerations.

### 3.5. Statistical Analysis

Statistical analysis was performed using GraphPad Prism software (version 8.02). The Shapiro–Wilk test was used to evaluate the normality of the HRA index distributions. Normally distributed data are presented descriptively with means and standard error of the mean (SEM). Group differences were assessed using a two-factor repeated-measures ANOVA with Holm–Sidak post hoc correction for multiple comparisons. *p*-values lower than 0.01 were considered statistically significant.

## 4. Results

The physiological and performance profiles of the male and female athletes showed notable differences. The mean age of participants in both groups was 19 ± 1 years, and significant gender-based differences were observed in key performance indicators. Male athletes demonstrated a mean maximal oxygen consumption (VO₂ max) of 67.5 mL/kg/min, a maximum power output of 355 watts, and a maximum heart rate (HRmax) of 204 beats per minute (bpm) (Table 1). In comparison, female athletes showed a mean VO₂ max of 58.5 mL/kg/min, a maximum power output of 265 watts, and a maximum heart rate of 194 bpm. These differences were statistically significant and are consistent with expected physiological distinctions between genders in terms of aerobic capacity and cardiovascular performance.

**Figure 1:**
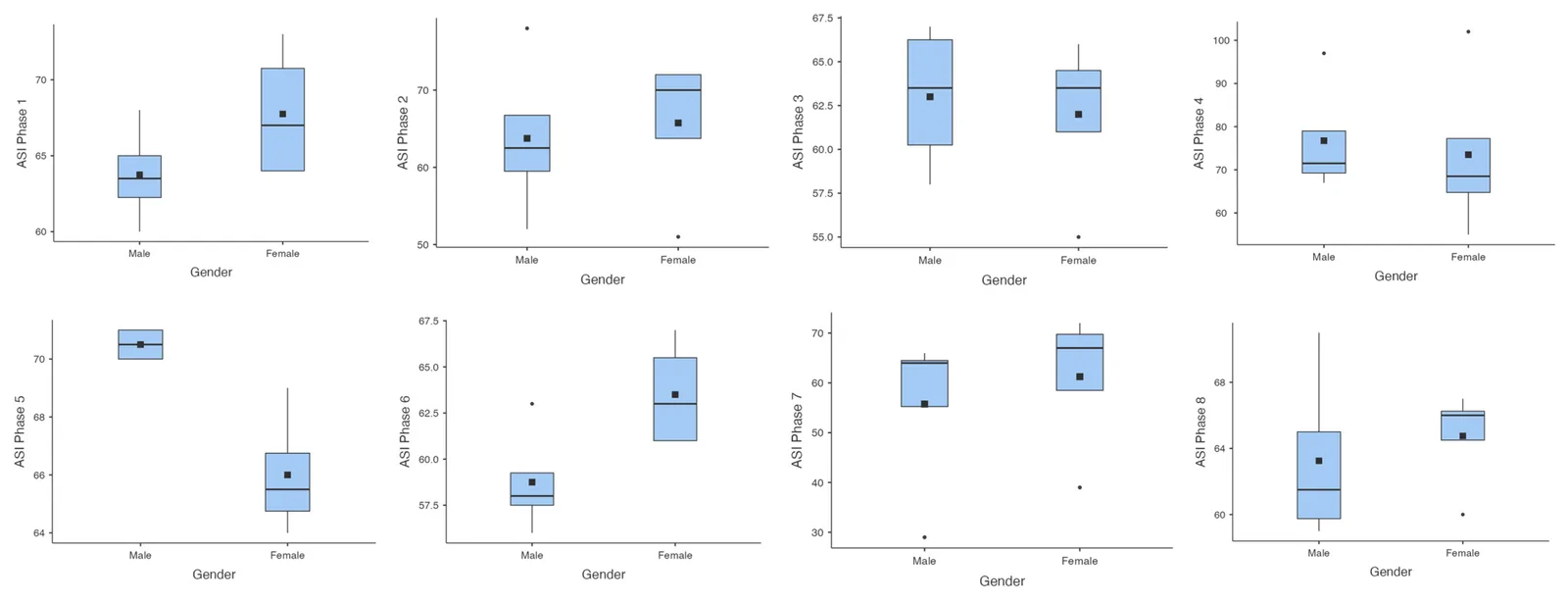
Asymmetric Spread Index of heart rate variability per phase. Note: A Kruskal–Wallis one-way ANOVA was used to compare ASI between genders across phases. A statistically significant difference was found only in Phase 5 (*χ*^2^(1) = 5.46, *p* = 0.019), indicating gender-related differences in asymmetric heart rate dynamics during this stage. The Asymmetric Spread Index (ASI) is unitless.

Regarding autonomic function, both male and female athletes exhibited normal patterns of autonomic modulation throughout the different phases of the 20 min test (see Table 2 and Table 3). Notably, after the postural change and exercise (Ruffier test) phases, there was a marked pattern of heart rate deceleration, indicating a strong vagal reactivation. This was followed by a symmetric pattern of acceleration, suggesting a balanced parasympathetic withdrawal during these transitions, as illustrated in Figure 1.

These autonomic responses reflect appropriate physiological adaptations to postural and physical stress in well-trained athletes. The observed deceleration pattern, particularly during recovery phases, may indicate efficient parasympathetic control, an important marker of cardiovascular fitness and autonomic flexibility.

The results demonstrate that while both groups showed healthy autonomic responses during the test, males exhibited significantly higher cardiorespiratory performance metrics, and both sexes showed phase-specific patterns of autonomic modulation, especially during transitions involving postural changes and exercise-induced stress.

Across the eight analyzed phases of the protocol, clear trends emerged in the short- and long-term heart rate variability (HRV) indicators when comparing male and female participants. The mean values of SD1, representing short-term parasympathetic activity, were consistently higher in males across all phases. This was especially evident during Phases 2 and 3, where male participants showed SD1 values of 126.80 ms and 163.70 ms, respectively, compared to 83.64 ms and 128.17 ms in females. These differences suggest a more prominent parasympathetic modulation or vagal rebound in males during early recovery phases.

Similarly, SD2, which reflects long-term variability and includes both sympathetic and parasympathetic components, also displayed higher values in males throughout the protocol. For instance, in Phase 2, males exhibited a mean SD2 of 171.70 ms, while that of females was 130.05 ms. The consistency of these findings across phases may indicate a more stable and adaptable autonomic profile in the male subgroup under the same physiological demands.

**Figure 2:**
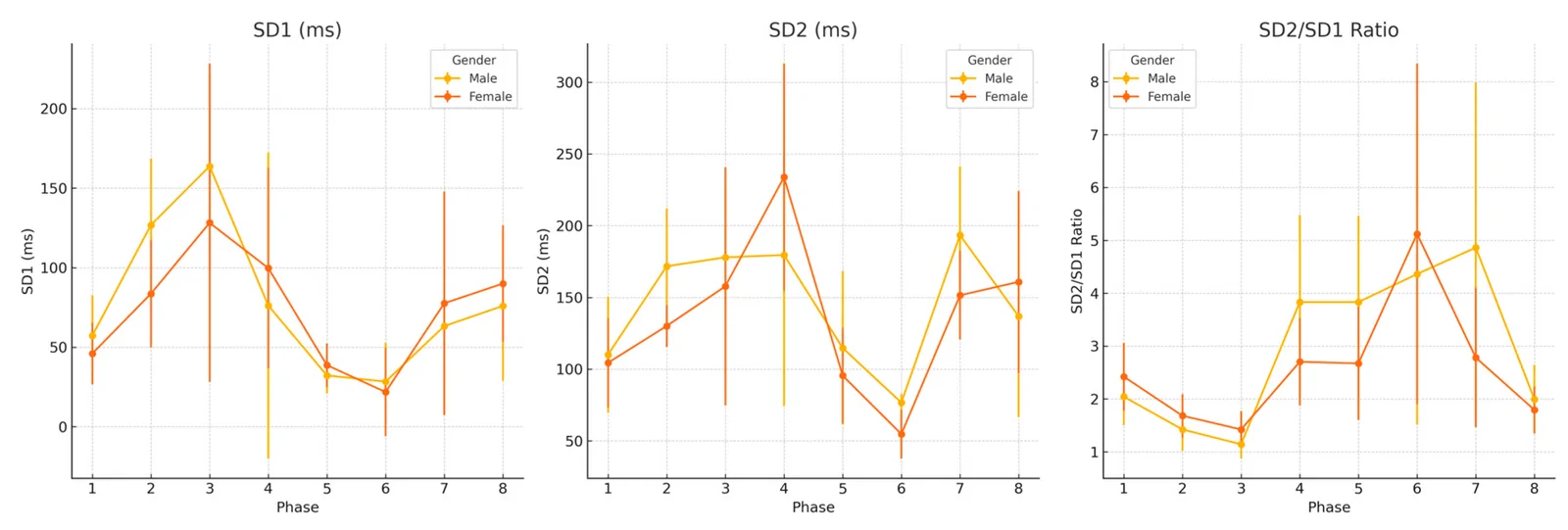
SD1, SD2, and SD1/SD2.

In contrast, the SD2/SD1 ratio, an indicator of the balance between long- and short-term variability (and often interpreted as a sympathovagal balance index), was slightly higher in females in most phases. For example, in Phase 1, the ratio was 2.42 in females versus 2.04 in males. This could reflect a comparatively stronger sympathetic influence or a reduced vagal rebound in the female participants during initial rest or recovery stages (see Figure 2).

**Figure 3:**
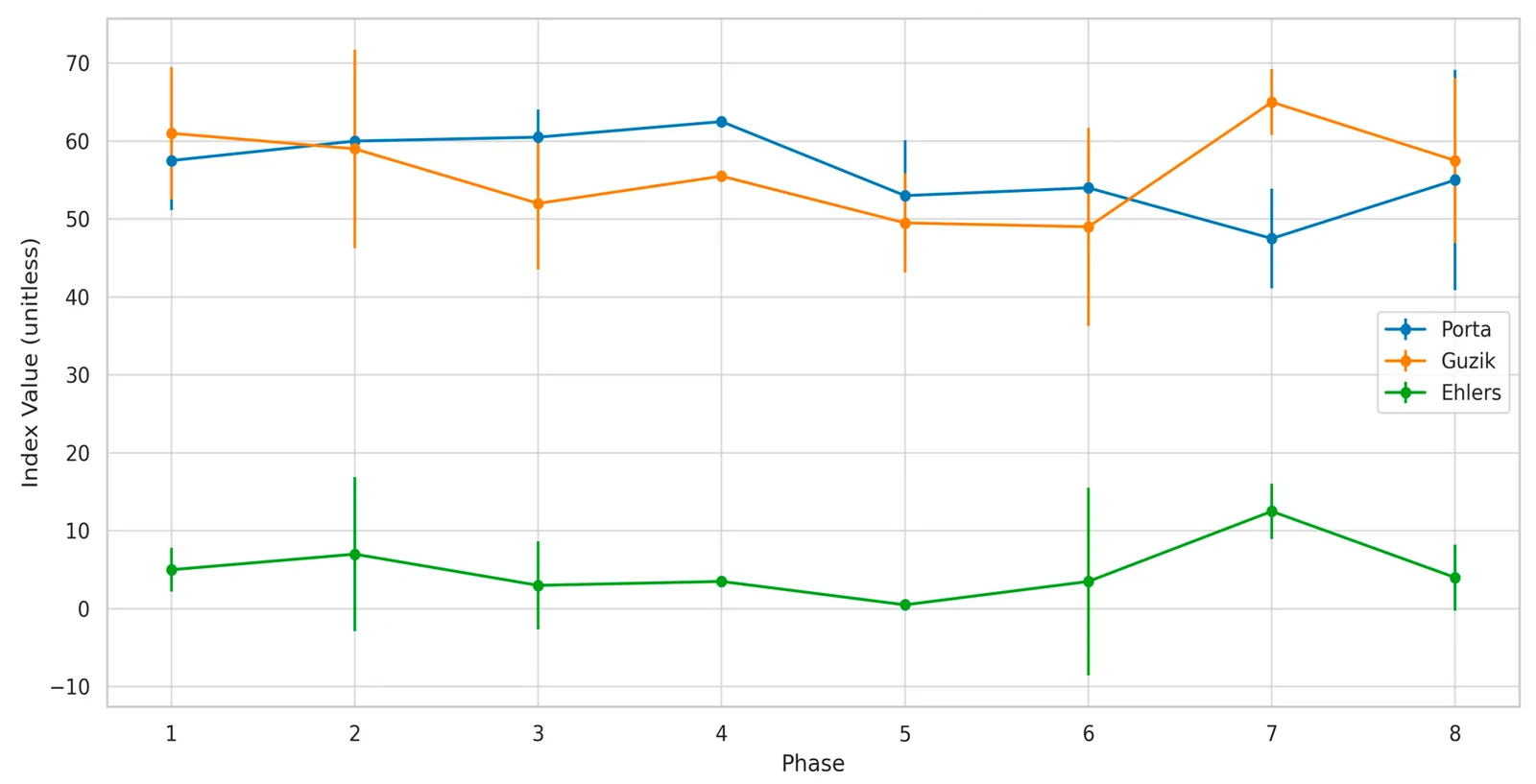
Index values per phase.

Figure 3 displays the evolution of the three heart rate variability (HRV) indices—Porta, Guzik, and Ehlers—across the eight phases of the protocol. In general, the Porta Index remains relatively stable across phases, with slight increases in Phases 2 and 4. This may reflect a higher presence of non-linear points in the Poincaré cloud, indicating increased autonomic complexity in those phases. The Guzik Index shows a rising trend in the early phases, peaking in Phase 4, suggesting increased sympathetic modulation or greater sympathetic–parasympathetic imbalance. The Ehlers Index shows the most fluctuation among the three, including negative values in Phases 6 and 4. These could indicate parasympathetic dominance or temporary autonomic asynchrony during those specific phases.

**Figure 4:**
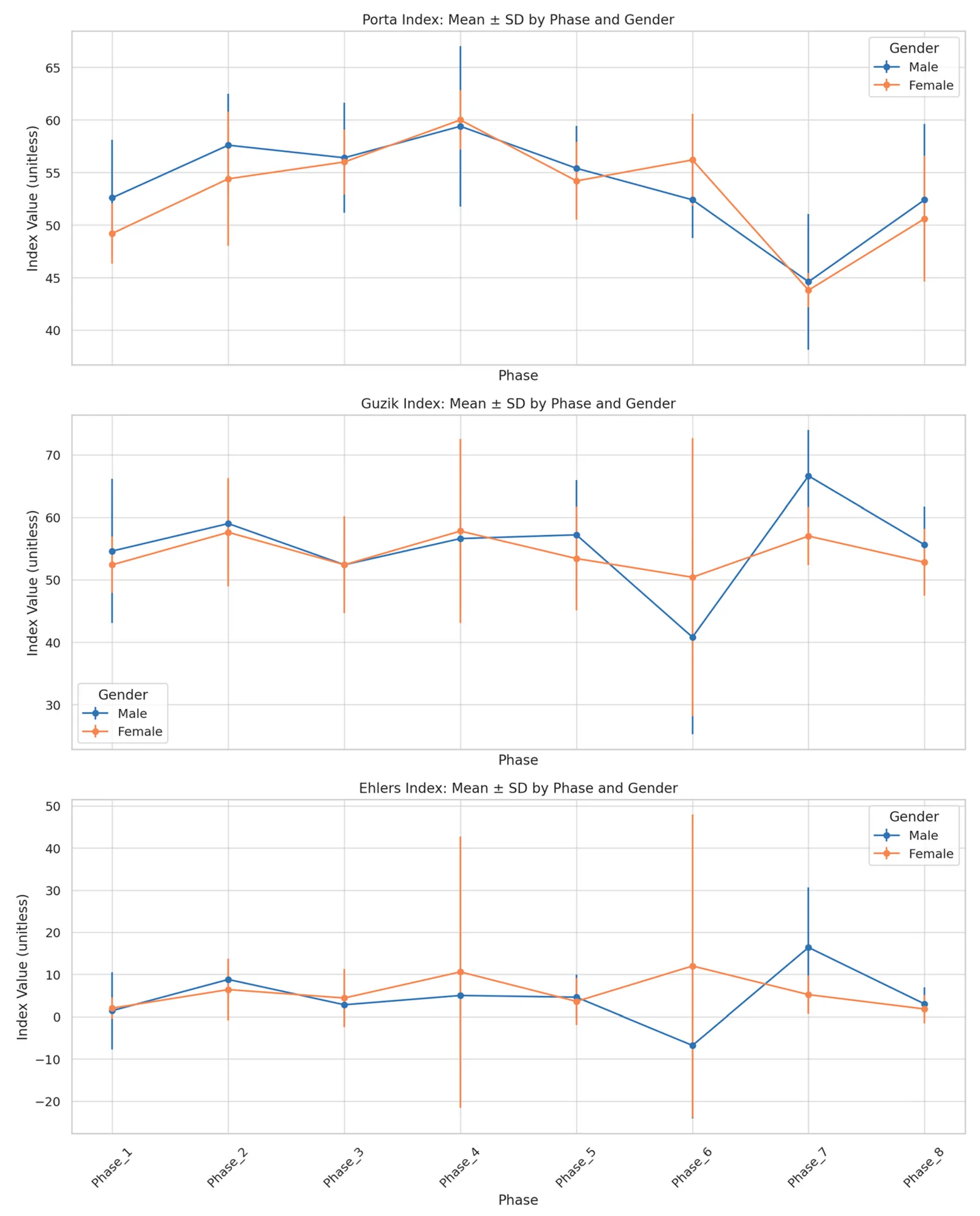
Indices by gender. Mean ± SD values of the Porta, Guzik, and Ehlers Indices across experimental phases, separated by gender (male and female). A two-way ANOVA revealed a significant main effect of the phase on the Porta Index (*p* < 0.001), but no significant effects of gender (*p* = 0.478) or the phase × gender interaction (*p* = 0.811). The Guzik Index showed a trend toward significance across all phases (*p* = 0.054), while the Ehlers Index showed no significant differences. Post hoc Tukey HSD tests for the Porta Index revealed that Phase 4 significantly differed from Phase 1 (*p* = 0.003) and Phase 8 (*p* = 0.008), and that Phase 7 was significantly lower than Phases 2 through 6 (all *p* < 0.001) and higher than Phase 8 (*p* = 0.027).

Figure 4 compares the average values (±standard deviation) of the three indices between females and males, phase by phase. The Porta Index tends to be slightly higher in females during the initial phases (1 to 3), although males show a notable increase in Phase 2, possibly reflecting stronger vagal reactivation. The Guzik Index is clearly higher in males during Phases 2 and 4, which may suggest heightened sympathetic activity or greater asymmetry in RR interval acceleration—potentially as a response to physical demand or stress. The Ehlers Index reveals negative values in female athletes during phases 4 and 6), while the male athletes’ values remain mostly positive, except in Phase 6. This index, associated with RR interval symmetry, might reflect transient autonomic instability in females during those stages.

## 5. Discussion

The main results of the present work showed the following: 1. Asymmetrical propagation of heart rate was observed, with a greater deceleration pattern after postural change and effort (Ruffier test) and a symmetrical acceleration pattern in these two phases. 2. Athletes exhibit increased vagal response. 3. Mountain bikers show rapid heart rate reductions after exertion. 4. Statistical differences in performance and neurovegetative balance based on gender were noticed.

### 5.1. Asymmetrical Propagation of Heart Rate

In the work on the physiology of MTB XC by Impellizzieri from 2007 [18], patterns of heart rate increase and decrease according to the intensity of the effort were described. This is consistent with our findings; however, the behavior of heart rate variability in the 20 min test and its acceleration and deceleration patterns were not considered in this previous study.

In the present analysis of heart rate variability (HRV) across eight structured phases of evaluation in physically active participants, characteristic patterns were observed, allowing for a direct comparison with recent scientific evidence. In general, the values of RMSSD and SD1—reflecting parasympathetic activity and short-term variability—peaked during Phases 2 and 3. This aligns with findings from several studies that identify these stages as periods of vagal recovery post-exercise or after moderate-intensity exertion. This trend is consistent with the observations reported by Solaro et al. (2023) [7], who evaluated Olympic athletes during a cycling exercise protocol and found greater amplitude in variability signals (interpretable as SD1 and RMSSD) during aerobic phases of effort, suggesting an efficient autonomic response to progressive workloads. Similarly, in the study by Swart and Constantinou (2023) [4] involving amateur cyclists in a three-day race, a progressive decline in RMSSD and SD1 was noted after each day of competition, reinforcing the usefulness of these indicators in detecting the cumulative impact of exercise on the autonomic nervous system.

Phase 4, in turn, showed a considerable increase in the SD2/SD1 ratio, suggesting sympathetic dominance or an imbalance in autonomic modulation. This observation is supported by the study by Wundersitz (2022) [19], who found that the LF/HF ratio (commonly associated with sympathovagal balance) increased significantly following a high-load exercise protocol and served as a strong predictor of post-exercise arrhythmias—especially when measured during deep sleep. In our analysis, the elevation of SD2/SD1 during the later stages (Phases 4 and 5) may reflect a transient autonomic stress response, a well-documented phenomenon in athletes following prolonged or intense activity. Additionally, the results revealed gender differences: male participants tended to show higher RMSSD and SD1 values compared to female participants in most phases. This could be attributed to physiological and hormonal differences affecting vagal tone, although the reviewed literature does not specifically delve into this comparison. Nevertheless, this sex-based difference could be of interest for future research.

Finally, regarding the bradycardic component that is often observed in athletes, the study by Molina et al. (2013) [20] found that although elite cyclists exhibited significantly lower resting heart rates, their HRV values did not differ substantially from those of an active control group. This suggests that bradycardia may be more related to intrinsic adaptations of the sinus node than to enhanced parasympathetic modulation. In contrast, the current results do reflect an active autonomic component, as indicated by increased vagal indices during early phases, followed by a relative sympathetic rise in intermediate phases, suggesting that autonomic control dynamically modulates heart rhythm according to the physiological demands of the protocol.

### 5.2. Vagal Response in Athletes

Grant, CC et al. (2012) performed exercise interventions in healthy young volunteers by increasing vagal influences during clinostatism, postural changes, and orthostatism maneuvers [21]. Gourine & Ackland (2019) affirmed that the frequent practice of sports activities generates a greater vagal activity compared to sedentary individuals [22], which is consistent with the present work. Mina Paz et al. (2022) described their analysis of heart rate asymmetry and the acceleration and deceleration behavior of heart rate variability in athletes and non-athletes [8,23], with results that were similar to ours in the group of athletes, although in the present work, the pattern of deceleration was greater after the postural change.

Pawłowski et al. (2021) [24] assessed the cardiovascular system’s response to orthostatic stress in a group of 133 healthy men, using heart rate asymmetry (AFC) methods during the initial phases of the head tilt test, specifically supine position and tilt. Several HRA calculation methods, such as the Porta Index (PI) and the Guzik Index (GI), along with variance-based components, were employed to analyze heart rate variability (HRV) and its asymmetry. The results showed a short-term HRA in 54.1% of men in the supine phase and in 65.4% in the inclined phase, with significant changes in the Guzik Index and HRV descriptors between the phases of the test. However, our study presented different phases of a test that included clinostatism, controlled ventilation at 10 cycles/min (VC10), controlled ventilation at 12 cycles/min (VC12), postural change, orthostatism, a Ruffier test, 1 min recovery, and final recovery. These phases allow for a more dynamic behavior of autonomic modulation due to the responses that the heart rate must have, putting its great asymmetry to the test; and it is important to mention that our sample was represented by the adaptations that athletes have to high performance.

### 5.3. Neurovegetative Balance in Mountain Bikers by Gender

Gender differences in heart rate variability (HRV) in mountain bikers are influenced by distinct autonomic and hormonal mechanisms. Koenig J & Thayer J. (2016) found that female athletes exhibit higher parasympathetic activity at rest, reflected in elevated RMSSD and high-frequency components [25]. This is due to Estrogen having been shown to enhance vagal tone, specially by the production of progesterone during the luteal phase. In contrast, male athletes have shown higher sympathetic activity during intense exercise. Our work shows similar findings, where females presented a vagal dominance during the supine phases (Phase 1 and Phase 8 of the test).

Solaro et al. (2023) showed that autonomic amplitude and oscillation indicators could differentiate between sports disciplines (e.g., cycling vs. shooting), revealing unique autonomic response profiles that might be interpreted as markers of physiological efficiency or training adaptation [20]. Similarly, HRV-derived indices such as the Porta, Guzik, and Ehlers Indices capture not only the sympathovagal balance but also reflect the fatigue status, risk of overtraining, and efficiency in post-exercise recovery [26].

Moreover, the relevance of sex differences in HRV responses has also been emphasized. Women tend to exhibit higher parasympathetic activity at rest, whereas men show a relatively higher sympathetic tone during physical exertion. This highlights the importance of conducting sex-specific analyses when using autonomic asymmetry indices to monitor athletic populations [26].

In addition, research by Özgünen et al. (2022) has shown that the evaluation of autonomic indices in conjunction with biomarkers such as troponins and creatine kinase (CK-MB) after exercise can provide critical information regarding cardiovascular stress and myocardial strain in athletes [27].

Finally, the implementation of advanced analytical models, such as Multi-Ethnic Factor Analysis (MEFA), helps integrate multiple autonomic indices, reduce data complexity, and enhance practical applications in both sports and clinical contexts [7]. This integration is crucial for accurately interpreting autonomic asymmetry indices and tracking their progression during different training and competition phases.

## 6. Conclusions

Mountain biking (MTBXC) athletes show a symmetrical acceleration to sympathetic stimuli such as postural changes and the Ruffier test, with a pattern of greater deceleration after these stimuli. More studies in high-performance athletes are needed to show asymmetric changes due to the adaptations of the autonomic modulation sensors.

## 7. Limitations

The present work had the fallowing limitations: the sample was limited by the performance level of the cyclists (National Team), which does not allow these results to be extrapolated to the general population.

## Figures and Tables

**Table 1:** Population.

	Male	Female
Age (yr)	19 ± 1	19 ± 1
Weight (Kg)	63.54	57.5
Height (cm)	171.8	160.2
BMI (kg/m^2^)	21.56	22.49
FAT (%)	8	13
VO_2max_ (mL/kg/min)	67.5	58.5
HR_max (bpm)_	204	194
Power_max_ (watts)	355	265

**Table 2:** Female athletes’ cardiovascular autonomic profile.

	HF (ms^2^) (Mean SD)	LF (ms^2^) (Mean SD)	VLF (ms^2^) (Mean SD)	LF/HF (Ratio) (Mean SD)	RMSSD (ms) (Mean SD)	PNN50 (%) (Mean SD)
Phase 1	2233.60 (2047.47)	1318.80 (1192.05)	395.80 (307.01)	0.75 (0.49)	79.22 (40.73)	48.90 (25.05)
Phase 2	9921.40 (4066.71)	3015.20 (4540.54)	346.40 (505.25)	0.24 (0.25)	127.26 (47.06)	51.02 (19.77)
Phase 3	17,864.20 (25,684.51)	4001.60 (5512.22)	1489.20 (3069.58)	0.22 (0.11)	165.86 (99.63)	56.24 (13.46)
Phase 4	3518.20 (1598.80)	5456.80 (2731.57)	2833.20 (1931.24)	1.62 (0.56)	135.36 (95.80)	23.92 (17.07)
Phase 5	689.40 (202.42)	1624.60 (765.58)	523.20 (426.36)	2.33 (0.88)	51.38 (18.53)	12.02 (8.83)
Phase 6	1347.00 (2977.40)	780.20 (1612.93)	164.80 (191.72)	2.97 (2.40)	29.76 (34.16)	1.46 (1.58)
Phase 7	1606.60 (1142.73)	972.80 (838.02)	240.00 (235.98)	0.60 (0.19)	88.26 (54.74)	22.78 (9.50)
Phase 8	2143.20 (1830.63)	1722.20 (1880.27)	363.40 (445.33)	0.81 (0.38)	112.02 (37.43)	51.68 (13.27)

**Table 3:** Male athletes’ cardiovascular autonomic profile.

	HF (ms^2^) (Mean SD)	LF (ms^2^) (Mean SD)	VLF (ms^2^) (Mean SD)	LF/HF (Ratio) (Mean SD)	RMSSD (ms) (Mean SD)	PNN50 (%) (Mean SD)
Phase 1	2114.60 (1662.04)	2645.80 (4096.01)	411.40 (512.80)	0.91 (0.74)	77.40 (36.35)	45.34 (24.33)
Phase 2	16,861.40 (12,485.93)	4810.00 (6040.46)	426.20 (645.18)	0.27 (0.24)	157.30 (72.17)	49.00 (26.92)
Phase 3	19,095.00 (20,116.92)	2668.20 (5018.58)	1257.60 (2608.54)	0.16 (0.18)	173.04 (115.90)	56.96 (27.43)
Phase 4	3165.20 (3616.31)	7213.40 (5844.94)	2727.00 (4501.18)	3.15 (1.22)	46.94 (24.71)	18.44 (10.82)
Phase 5	882.20 (864.23)	3595.80 (2172.76)	2193.80 (2397.88)	9.91 (11.71)	57.36 (40.62)	16.90 (16.80)
Phase 6	1464.00 (3255.16)	824.80 (1765.09)	169.40 (285.28)	3.91 (6.98)	56.74 (37.73)	7.34 (3.93)
Phase 7	4411.20 (8308.09)	1437.40 (2244.24)	364.00 (344.49)	0.53 (0.23)	103.02 (76.49)	18.12 (13.19)
Phase 8	3589.00 (3776.50)	3347.80 (3813.98)	388.00 (508.38)	0.83 (0.32)	113.10 (62.83)	47.20 (16.02)

## Data Availability

The original contributions presented in this study are included in the article. Further inquiries can be directed to the corresponding author.
